# Chloroplast dismantling in leaf senescence

**DOI:** 10.1093/jxb/erab200

**Published:** 2021-05-07

**Authors:** Fernando Domínguez, Francisco Javier Cejudo

**Affiliations:** Instituto de Bioquímica Vegetal y Fotosíntesis, Universidad de Sevilla and Consejo Superior de Investigaciones Científicas, Avda. Américo Vespucio 49, 41092-Sevilla, Spain; Instituto de Bioquímica Vegetal y Fotosíntesis, Universidad de Sevilla and Consejo Superior de Investigaciones Científicas, Avda. Américo Vespucio 49, 41092-Sevilla, Spain; Estación Experimental del Zaidín, CSIC, Spain

**Keywords:** Autophagy, chlorophagy, chloroplast, gerontoplast, plastoglobule, ROS, senescence

## Abstract

In photosynthetic plant cells, chloroplasts act as factories of metabolic intermediates that support plant growth. Chloroplast performance is highly influenced by environmental cues. Thus, these organelles have the additional function of sensing ever changing environmental conditions, thereby playing a key role in harmonizing the growth and development of different organs and in plant acclimation to the environment. Moreover, chloroplasts constitute an excellent source of metabolic intermediates that are remobilized to sink tissues during senescence so that chloroplast dismantling is a tightly regulated process that plays a key role in plant development. Stressful environmental conditions enhance the generation of reactive oxygen species (ROS) by chloroplasts, which may lead to oxidative stress causing damage to the organelle. These environmental conditions trigger mechanisms that allow the rapid dismantling of damaged chloroplasts, which is crucial to avoid deleterious effects of toxic by-products of the degradative process. In this review, we discuss the effect of redox homeostasis and ROS generation in the process of chloroplast dismantling. Furthermore, we summarize the structural and biochemical events, both intra- and extraplastid, that characterize the process of chloroplast dismantling in senescence and in response to environmental stresses.

## Teaser

Chloroplast dismantling is a complex process involving intra- and extraplastid events essential for remobilizing metabolic intermediates in senescence, and for eliminating damaged plastids caused by stressful environmental conditions.

## Introduction

Photosynthesis, the process that allows the conversion of sunlight into chemical energy, is essential for life on Earth. Light-driven photochemical reactions constitute the primary source of the organic material and oxygen that support the biological activity of heterotrophic organisms; for example, it is estimated that photosynthetic reactions produce 10 times more than the global consumption of primary organic material by humans (Nelson and Junge, 2015). In plants, oxygenic photosynthesis occurs in specialized organelles, the chloroplasts, which perform a cascade of energy conversions including light absorption by pigment cofactors, excitation energy transfer among antennae, electron transfer within and between photosystems (PS), establishment of an electrochemical proton gradient at the thylakoid membrane coupled to ATP synthesis, and generation of reducing equivalents in the forms of reduced ferredoxin and NADPH. Both reducing equivalents and ATP are subsequently used for carbon, nitrogen, and sulfur assimilation that generates the numerous metabolic intermediates produced in chloroplasts, such as amino acids, fatty acids, and purine and pyrimidine bases, among others. Chloroplasts are also the source of different plant hormones, emphasizing the important signalling function of the organelle. Chloroplasts are members of an extremely dynamic family of organelles, the plastids, whose forms interconvert into each other depending on developmental and environmental cues. The different types of plastids include proplastids and etioplasts, at early stages of plant development, pigment-bearing plastids (chromoplasts and chloroplasts), storage-specialized plastids (amyloplasts, elaioplasts and leucoplasts), and gerontoplasts, which are associated with senescence (Jarvis and López-Juez, 2013; Liebers *et al.*, 2017).

Most of the photosynthetic activity of plants is performed in leaves. Leaf development could be divided into two global phases: expansion, which is started by an initial stage of cell proliferation followed by a post-mitotic cell expansion stage that generates the mature leaf, and senescence, which extends from maturity to death. Both phases are characterized by sink-to-source transitions, so that leaf senescence allows the recycling of the components accumulated during growth and maturation into exportable nutrients to support the requirements of newly developing organs (Avila-Ospina *et al.*, 2014). In fact, the global sink-to-source transition encloses different partial transitions, which are highly coordinated. These include the metabolic transition from anabolic, based on photosynthesis and carbon assimilation during leaf expansion, to catabolic, characterized by the degradation of chlorophyll and other macromolecules during leaf senescence. There is a hormonal transition in which gibberellic acid and cytokinin responses operate until leaf maturity, whereas jasmonic acid (JA), abscisic acid (ABA), and salicylic acid responses operate in leaf senescence. Finally, it could also be considered a regulatory network transition, mainly involving the NAC and WRKY transcription factor families that regulate the expression of senescence-associated genes (SAGs) (Woo *et al.*, 2019).

Global approaches based on transcriptomic, proteomic, and metabolomic analyses have allowed establishment of the relationship between macromolecule degradation processes during leaf senescence, such as chlorophyll breakdown, proteolysis, lipid catabolism, and cell wall disassembly. These degradative processes are coordinated with nutrient remobilization events including the accumulation of γ-aminobutyric acid, branched-chain and aromatic amino acids or increase of ceramides, triacylglycerols, and polyols (Buchanan-Wollaston *et al.*, 2003, 2005; Breeze *et al.*, 2011; Watanabe *et al.*, 2013; Li *et al.*, 2017b; Sekhon *et al.*, 2019; Tamary *et al.*, 2019). From the quantitative point of view, chloroplasts contain several of the most abundant biological macromolecules on Earth. In this regard, Rubisco, light-harvesting complex II (LHCII), chlorophylls, and monogalactosyl diacylglycerol (MGDG) are, respectively, the most abundant soluble protein, membrane protein, pigment, and lipid in the biosphere (Kirchhoff, 2019). Thus, chloroplasts are key organelles that act as factories of metabolic intermediates to support plant growth, but also an important reservoir of nutrients for remobilization and recycling during senescence. The recycling function of chloroplasts constituents has a deep impact from a global point of view; for example, it is estimated that more than 10 billion tons of Rubisco and 1 billion tons of chlorophyll are degraded every year (Hendry *et al.*, 1987; Otegui, 2018).

Based on their capacity to serve as factories and reservoirs of metabolic intermediates, chloroplasts may be considered as central hubs during leaf development, coordinating the multi-level perception of signals, regulating the fluxes of energy and nutrients, and modulating the appropriate spatio-temporal responses, which include the participation in their own elimination during senescence and in response to stressful environmental conditions. Chloroplast dismantling is a complex process, which may proceed via different pathways. The turnover of chloroplast components provides nutrients for sink tissues and may be relevant to controlling the progression of senescence. Some of the degradation by-products of chloroplast dismantling are potentially harmful; thus, the process needs to be tightly controlled. Finally, chloroplast degradation involves intra- and extraplastid events, a multifactorial scenario that makes its analysis a rather complex issue.

In this review, we discuss the effect of chloroplast redox homeostasis and reactive oxygen species (ROS) as relevant upstream signals in the regulation of chloroplast dismantling. Moreover, we summarize the multiple features of chloroplast dismantling and their relationship with leaf senescence and response to stress. Finally, we describe the macromolecular rearrangements, targets of degradation and intra- and extraplastid breakdown pathways occurring during chloroplast dismantling.

## Redox regulation as a component of chloroplast dismantling

The photosynthetic electron transport chain operates in the presence of oxygen; thus, chloroplasts inevitably generate ROS as a by-product of photosynthesis. To avoid the detrimental effects of high levels of ROS, chloroplasts are equipped with antioxidant systems (Waszczak *et al.*, 2018); however, different environmental conditions provoke the imbalance between ROS production and scavenging, which generates oxidative stress. In addition, the signalling function of ROS as second messengers is well established, and is essential for plant development and response to environmental stimuli (Mittler, 2017). Leaf senescence is one of the developmental programmes modulated by environmental signals, in which ROS play an active role. Thus, chloroplasts, which constitute an important source of ROS in photosynthetic plant cells, but are also targets of ROS-triggered damage, are key organelles in this developmental programme.

Among the plant mechanisms of response to ever-changing environmental conditions, redox regulation based on dithiol–disulfide exchange plays an essential role (Buchanan, 2016; Cejudo *et al.*, 2019). Chloroplasts are the organelles equipped with the most complex redox network in plant cells including up to 20 thioredoxins (Trxs) or Trx-like proteins (for recent reviews see Geigenberger *et al.*, 2017; Cejudo *et al.*, 2019; Zaffagnini *et al.*, 2019). These Trxs are reduced by photo-reduced ferredoxin (Fdx) in a reaction catalysed by a Fdx-dependent Trx reductase, which links the redox regulation of chloroplast metabolism to light (Schürmann and Buchanan, 2008). In addition, chloroplasts harbour an NADPH-dependent Trx reductase (NTRC) with a joint Trx domain, which uses NADPH as source of reducing power (Serrato *et al.*, 2004; Bernal-Bayard *et al.*, 2012). It was recently shown that the function of these redox pathways is integrated via the redox balance of the hydrogen peroxide scavenging enzyme 2-Cys peroxiredoxin (Pérez-Ruiz *et al.*, 2017). Thus, thiol-dependent antioxidant and redox regulatory systems are functionally interconnected (Cejudo *et al.*, 2021), and may affect chloroplast stability in senescing leaves and in response to stress. In this regard, Arabidopsis mutants severely impaired in chloroplast redox homeostasis, such as those devoid of NTRC and *f*- or *x-*type Trxs, show high mortality at the seedling stage and bleaching of cotyledons, which are characterized by chloroplasts with the structural features of gerontoplasts (Ojeda *et al.*, 2017*a*, *b*).

These results suggest that chloroplast stability is affected by the redox homeostasis of the organelle. In support of this notion, plants that overexpress the protein disulfide isomerase/reductase AtCYO1 show decreased rates of chlorophyll degradation and disassembly of PSI and PSII supercomplexes, hence maintaining thylakoid integrity and delaying dark-induced senescence (Tominaga *et al.*, 2018). Moreover, the overexpression of AtCYO1 has a positive effect on the stability of stromal proteins, such as Rubisco, Fdx-NADPH reductase and Trx *m* (Tominaga *et al.*, 2018). It was proposed that AtCYO1 in its preferential localization to thylakoid membranes counteracts the oxidation of thiols of proteins in PS complexes, thereby interfering with the reactions of chlorophyll catabolic enzymes. The smaller fraction of the protein localized at the stroma could favour the reduction of Rubisco, hence explaining the maintenance of chloroplast integrity and stay-green phenotype during senescence of AtCYO1 overexpressing plants (Tominaga *et al.*, 2018).

## Plastid-to-nucleus retrograde signalling in senescence

The above-mentioned stay-green phenotype of AtCYO1 overexpressing plants (Tominaga *et al.*, 2018) suggests that the redox state of the chloroplast constitutes a relevant signal regulating the progression of the developmental programme of senescence. In line with this notion, mutants impaired in chlorophyll catabolic enzymes also display stay-green phenotypes (Oh *et al.*, 2003; Kusaba *et al.*, 2007; Park *et al.*, 2007; Sato *et al.*, 2009; Huang *et al.*, 2013; Yamatani *et al.*, 2013), further supporting the signalling function of chloroplasts in leaf senescence. ROS are clear candidates to act as signals derived from chloroplasts for the regulation of senescence since these organelles constitute the major source of ROS production in plant cells. Indeed, it is well-known that the increase of ROS favours cell death and leaf senescence (Quirino *et al.*, 2000). The finding that tobacco plants deficient in photosynthetic NAD(P)H dehydrogenase (NDH), and hence with decreased chloroplast ROS production, show delayed senescence (Zapata *et al.*, 2005) is an additional indication of the role of the redox state of these organelles on senescence.

There is extensive evidence connecting ROS and autophagy (Pérez-Pérez *et al.*, 2012). ROS and the redox regulatory network of the chloroplast modulate the oxidation state of cysteine residues, which may affect the progression of senescence and the response to environmental cues. However, the knowledge of redox-regulated targets in chloroplast dismantling is still poor. Some of the proteins identified as targets of Trxs (Montrichard *et al.*, 2009), NTRC (Yoshida and Hisabori, 2016; González *et al.*, 2019), and 2-Cys peroxiredoxins (Cerveau *et al.*, 2016; Liebthal *et al.*, 2020) have also been identified as critical targets for chloroplast degradation during senescence or in response to environmental stresses. Thus, these targets might participate in redox regulation of chloroplast dismantling, but more work is needed to address their role in the process.

The mechanisms for chloroplast quality control in response to environmental conditions that cause oxidative stress allow damaged chloroplasts to activate their own degradation. Two major pathways were proposed to target damaged chloroplast for degradation in the central vacuole (Woodson, 2019). In the first pathway, chloroplasts damaged by singlet oxygen accumulation undergo ubiquitination of envelope proteins by the cytoplasmic E3 ubiquitin ligase PUB4 (Woodson *et al.*, 2015). Then, ubiquitinated proteins are recognized (probably through a putative adaptor protein) by the ATG machinery and the tonoplast membrane of the central vacuole. In this case, ubiquitin-marked plastids show a great internal breakdown prior to the release into the vacuole. Finally, vacuolar proteases fulfil the cleavage and turnover of plastid components (Woodson *et al.*, 2015). In the second pathway, chloroplasts damaged by excess light or UV-B radiation are targeted for degradation (Woodson, 2019). Chloroplasts damaged by accumulation of superoxide and H_2_O_2_ in response to UV-B are targeted to the central vacuole in an ATG-dependent manner (Izumi *et al.*, 2017). More recently, it was shown that a double mutant of Arabidopsis affected in autophagy and PUB4 showed accelerated chlorosis linked to ROS accumulation in senescing leaves indicating the combined action of chloroplast autophagy and ubiquitination in the response to starvation and oxidative stress (Kikuchi *et al.*, 2020).

## Chloroplast macrodomain and subdomain rearrangements during leaf senescence

**Fig. 1.**
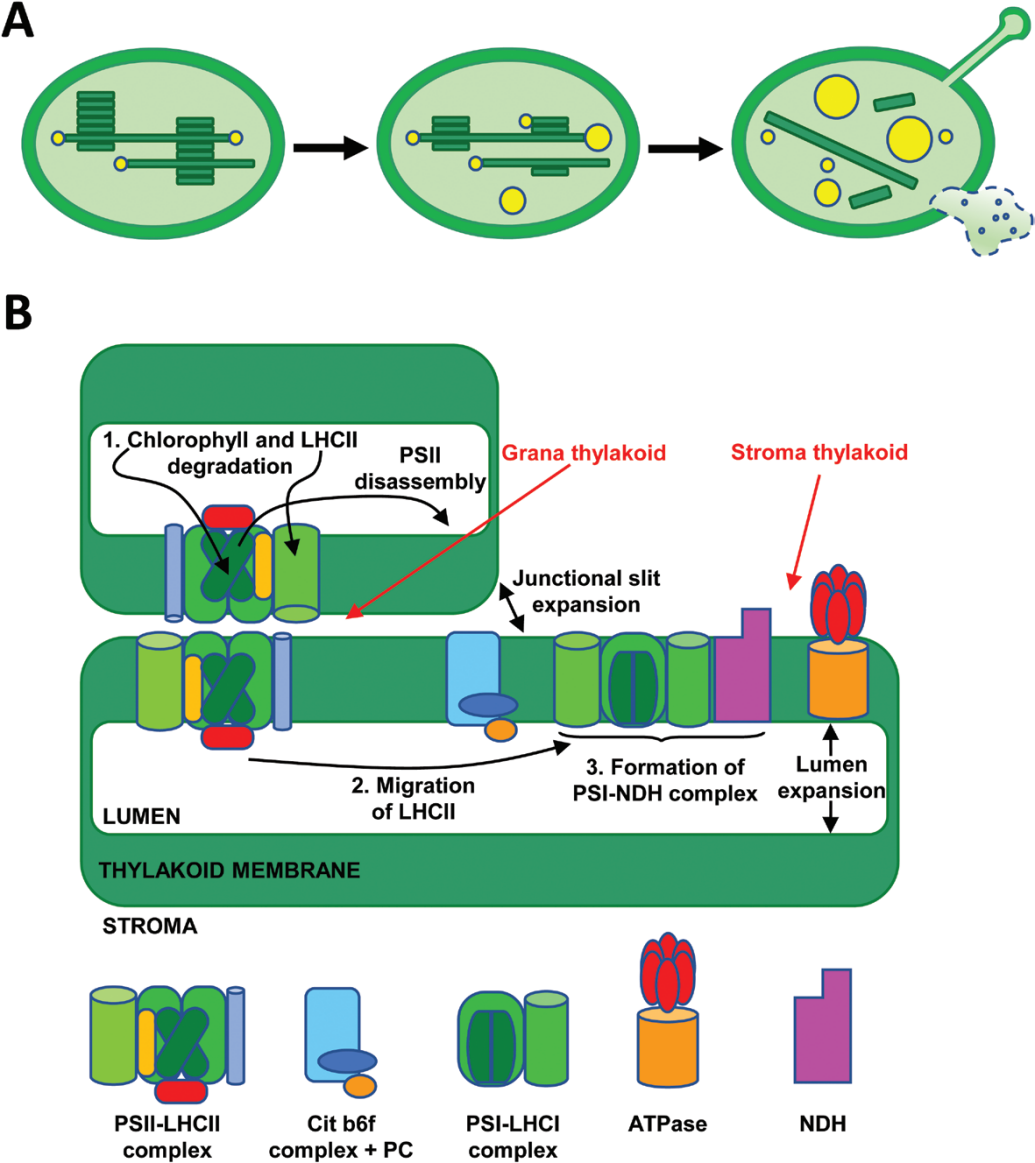
Chloroplast macrodomain and subdomain rearrangements during senescence. (A) Main ultrastructural changes associated with chloroplast-to-gerontoplast transition. As senescence progresses, mature chloroplasts (left panel) show increased disorganization of the thylakoid membrane system and an increase in the size and number of plastoglobules (middle panel); finally, gerontoplasts (right panel) are characterized by the almost complete loss of thylakoids, highest plastoglobule size, and envelope perforations that allow extrusion of stroma content. (B) Rearrangements occurring during grana unstacking in the chloroplast-to-gerontoplast transition were classified in stages: 1, chlorophyll and LHCII degradation; 2, LHCII migration; 3, formation of PSI–NDH complex. Schemes representing each of the complexes are indicated.

Although chloroplasts suffer changes very early in the onset of leaf senescence, these organelles are the last to collapse during this developmental programme (Lim *et al.*, 2007). The most characteristic morphological features of chloroplast degeneration, hence the transition of chloroplast to gerontoplast, are volume alterations and the transition from ellipsoid to circular morphology, which are combined with a deep reorganization of internal membranes. Mulisch and Krupinska (2013) identified three stages as the most relevant ultrastructural changes associated with the transition of mature chloroplast to gerontoplast: breakdown of the thylakoid membrane system, increase in size and number of plastoglobules, and alteration and disruption of the plastid envelope, which are schematized in Fig. 1A.

The disorganization of the internal membrane network of the chloroplast is characterized by grana unstacking, flattening of thylakoids, swelling of intrathylakoid space, and finally, degradation of stroma lamellae. This sequential process can occur with multiple variants during the progress of senescence, depending on the plant species, environmental conditions, and stress factors (reviewed by Mulisch and Krupinska, 2013). For example, different types of gerontoplasts were observed in the barley cultivars Carina and Lomerit during senescence under field conditions, which reflect alternative strategies of chloroplast dismantling with consequences for crop yields (Krupinska *et al.*, 2012). Anomalous breakdown of the thylakoid membrane system may evolve by the formation of dilations at the thylakoid ends, cup-shape stacked membranes, and thylakoid coiling (Wrischer *et al.*, 2009).

Associated with these rearrangements of the internal membrane network, the structural changes characteristic of gerontoplasts include macromolecular reorganization of thylakoid protein complexes, which can be subdivided into three stages, as summarized in Fig. 1B. Grana stacking depends on the mutual interactions between PSII–LHCII supercomplexes facing each other in adjacent thylakoid membranes (Albanese *et al.*, 2020). Reverse genetic studies have shown that dismantling of PSII–LHCII supercomplex, degradation of the LHCII, and breakdown of chlorophyll are prerequisites for grana unstacking (Fig. 1B, stage 1). In *Cucumis* cotyledons, it was shown that senescence induces changes in the photochemical apparatus of chloroplasts consisting in the migration of LHCII from stacked grana thylakoid to stroma lamellae and its association with PSI, which might cause typical unstacking and flattening of the grana structure (Prakash *et al.*, 2001, 2003) (Fig. 1B, stage 2). In other plants, such as spinach, tobacco, or Arabidopsis, the formation of PSI–LHCII megacomplexes in senescing chloroplasts was also observed (Schwarz *et al.*, 2018). The remobilization of nutrients, dismantling of photochemical machinery, and disruption of linear electron transport are main events during senescence; however, gerontoplasts undergo additional macromolecular rearrangements. These rearrangements allow alternative electron transport pathways that preserve the production of ATP necessary for attending these highly energy demanding processes (Krieger-Liszkay *et al.*, 2019). Thus, total or partial loss of PSII and/or PSI complexes occurring in senescence may be compensated by the induction of NDH and Plastid Terminal Oxidase complexes, which enhance cyclic (Zapata *et al.*, 2005) or chlororespiratory electron flow (Tallón and Quiles, 2007), as alternative electron transport pathways (Fig. 1B, stage 3).

There are numerous regulatory processes of photosynthesis that are closely linked to the dynamics of the thylakoid membrane. These processes include the repair cycle of PSII, photoprotective energy dissipation, state transitions, and alternative electron transfer pathways (Ruban and Johnson, 2015; Yoshioka-Nishimura, 2016). The main structural changes of thylakoid membranes are reversible grana stacking and unstacking, the dynamics of which depends on the increase of membrane fluidity, the thylakoid lumen expansion, and the enlargement of the junctional slits between adjacent thylakoids (Fig. 1B). CURVATURE THYLAKOID1 proteins are involved in such membrane dynamics through their phosphorylation, oligomerization, and thylakoid curvature-induced movements at grana margins (Armbruster *et al.*, 2013; Pribil *et al.*, 2018; Trotta *et al.*, 2019). However, harsh photo-oxidative conditions promote irreversible thylakoids unstacking (Khatoon *et al.*, 2009).

**Fig. 2.**
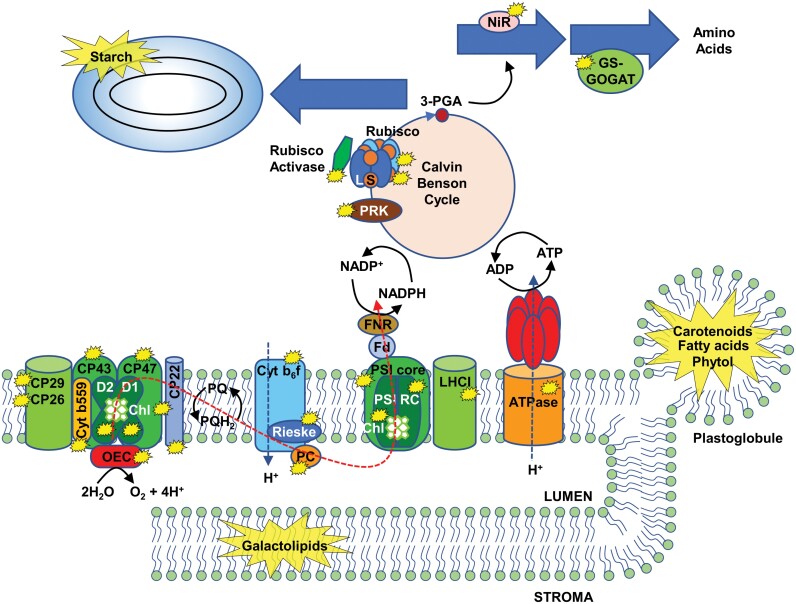
Localization of the main targets undergoing degradation during chloroplast dismantling. Thylakoid membrane and stromal components degraded during chloroplast dismantling are marked with yellow stars. Red dashed line indicates the linear flow of the photosynthetic electron chain. Cyt, cytochrome; Fd, ferredoxin; FNR, reduced ferredoxin-NADPH reductase; GOGAT, glutamate synthase; GS, glutamine synthase; LHCI, light-harvesting complex I; NIR, nitrite reductase; OEC, oxygen-evolving complex; PC, plastocyanin; 3-PGA, 3-phosphoglyceric acid; PRK, phosphoribulokinase; PSI, photosystem I; PSIRC, PSI reaction centre.

The second ultrastructural hallmark characterizing the chloroplast-to-gerontoplast transition is the increased number and size of plastoglobules (Mulisch and Krupinska, 2013) (Fig. 1A). Plastoglobules are lipid droplets surrounded by a lipid monolayer, which are attached to the thylakoid membranes and remain contiguous to their outer side (Austin *et al.*, 2006) (Fig. 2). Different environmental stressful conditions, including oxidative stress, high light intensity, nitrate starvation, drought, high salinity, viral infection, chilling, and ozone, as well as developmental programmes, such as senescence and fruit development, determine the remobilization of thylakoid membranes and the subsequent increase in the number and size of plastoglobules due to the lipid accumulation in their hydrophobic core (Besagni and Kessler, 2013). In such circumstances, plastoglobules form grape-like clusters that are attached to each other and remain continuous by extensions of the half-lipid bilayer (Austin *et al.*, 2006).

Chloroplast double-membrane envelopes usually show alterations and disruptions during senescence, which include perforations, broken envelope (Springer *et al.*, 2016) and the formation of stromules (Ishida *et al.*, 2008). As an example, virus-induced senescence is characterized by chloroplast malformations such as membrane-bound extrusions, ameboid morphology, cytoplasmic invaginations, and generation of stromules (Zhao *et al.*, 2016). Stromules are observed in all types of plastids at any developmental stage; however, these structures are more abundant under different conditions, such as high levels of ROS or sugar, and in senescence-related processes (Brunkard *et al.*, 2015; Caplan *et al.*, 2015). It has been suggested that stromules participate in the formation and releasing of Rubisco-containing bodies (RCBs) (Ishida *et al.*, 2008) and small starch granule-like (SSGL) bodies (Wang *et al.*, 2013), two of the extraplastid pathways for chloroplast degradation that will be discussed below. Moreover, stromules may surround the nucleus and endoplasmic reticulum, which suggests their role in chloroplast-to-nucleus retrograde signalling during senescence and in transport of pro-defence signals into the nucleus during innate immunity (Schattat *et al.*, 2011; Caplan *et al.*, 2015; Hanson and Hines, 2018).

## Sequential degradation of chloroplast components

The different reorganization events and structural changes occurring during chloroplast dismantling are associated with the cleavage of the most relevant constituents (pigments, proteins, lipids, and starch) of the organelle, which are summarized in Fig. 2. A more detailed description of the degradation of each of these types of molecules follows.

### Pigments

Chlorophyll breakdown is one of the most important events of chloroplast degradation in senescent leaves, so yellowing appears as a clear symptom of senescence. Up to six enzymes participate in the multi-step process of chlorophyll cleavage, which is tightly regulated (Hörtensteiner and Kräutler, 2011). Different phases may be identified in the process: (i) the two-step reduction of chlorophyll *b* to chlorophyll *a* catalysed by chlorophyll *b* reductase (Kusaba *et al.*, 2007; Sato *et al.*, 2009) and 7-hydroxymethyl-chlorophyll *a* reductase (Meguro *et al.*, 2011); (ii) removal of the central Mg atom of chlorophyll *a* catalysed by Mg-dechelatase to generate pheophytin *a* (Shimoda *et al.*, 2016); (iii) elimination of the phytol chain of pheophytin *a* by pheophytinase that produces pheophorbide *a* (Schelbert *et al.*, 2009); and (iv) the two-step formation of non-phototoxic primary blue-fluorescent chlorophyll catabolite by pheophorbide *a* oxygenase (Pruzinská *et al.*, 2003) and red chlorophyll catabolite reductase (Pruzinská *et al.*, 2007). Based on evidence indicating that all the chlorophyll catabolic enzymes interact directly or indirectly with each other, it has been suggested that these enzymes act to form a metabolic channel. This would facilitate the breakdown of the pigment at LHCII, hence allowing a tight control of phototoxic chlorophyll cleavage by-products (Sakuraba *et al.*, 2012).

**Fig. 3.**
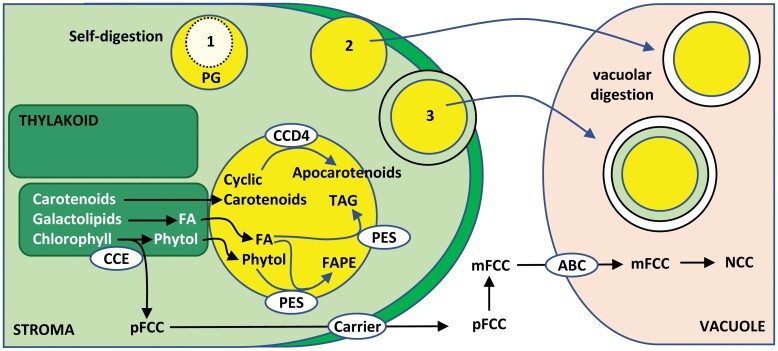
Intra- and extraplastid pathways for pigment and lipid degradation during chloroplast dismantling. Chlorophyll and galactolipid breakdown are initiated within thylakoid membranes. Phytol and fatty acids, by-products in the cleavage of chlorophyll and galactolipids, respectively, are esterified in plastoglobules (yellow) to avoid their toxicity. The first non-toxic, colourless by-product of chlorophyll breakdown (pFCC) is released to the chloroplast stroma and then to the cytosol where it is converted to modified fluorescent chlorophyll catabolite (mFCC), which is transported to the vacuole by an ATP-binding cassette (ABC)-type vacuolar transporter. In the vacuole, mFCC is degraded to non-fluorescent chlorophyll catabolite (NCC). Alternative types of plastoglobules elimination are indicated with numbers: 1, self-digestion within chloroplast; 2, vacuolar degradation of extruded plastoglobule; 3, plastoglobule-containing vesicles after microautophagy. CCD4, carotenoid cleavage dioxygenase 4; FA, fatty acid; FAPE, fatty acid phytyl esters; PES, phytyl ester synthase; pFCC, primary blue-fluorescent chlorophyll catabolite; TAG, triacylglycerol.

Phytol released by the pheophytin pheophorbide hydrolase-mediated chlorophyll cleavage pathway and free fatty acids resulting from the degradation of galactolipids of the thylakoid membranes accumulate inside plastoglobules, thereby avoiding their toxicity (Besagni and Kessler, 2013; Rottet *et al.*, 2015). In plastoglobules, fatty acids and phytol are converted to fatty acid phytyl esters, tocopherol and triacylglycerol by the multifunctional plastoglobule-localized phytyl esters synthases PES1 and PES2, and by a third member of the esterase/lipase/thioesterase family (Lippold *et al.*, 2012; Vom Dorp *et al.*, 2015) (Fig. 3). Carotenoid degradation seems to occur also in plastoglobules since carotenoid cleavage dioxygenase 4 (CCD4) appears as a major component of these structures (Ytterberg *et al.*, 2006; Lundquist *et al.*, 2012; Rottet *et al.*, 2016). The plastoglobule-localized Zn-protease PGM48 has been proposed as a regulator of PES1, PES2, and CCD4 enzymes (Bhuiyan *et al.*, 2016). Recent proteomic analyses revealed the presence of up to 30 proteins in plastoglobules, including SOUL4 (Lundquist *et al.*, 2012), a protein that has been shown to have haem binding activity (Shanmugabalaji *et al.*, 2020). Since haem accumulation may increase ROS production, the haem-binding activity of SOUL4 suggests an additional role for plastoglobules in controlling the chloroplast pool of haem groups, hence avoiding potential harmful effects of ROS production by these groups.

### Proteins

Senescence-associated chlorophyll breakdown and the disruption of linear electron flow suggest the progressive dismantling of photosynthetic supercomplexes. Western blotting and blue-native polyacrylamide gel electrophoresis have allowed the deciphering of major changes in thylakoid supercomplexes and protein alterations during senescence, which are summarized in Fig. 2. After chlorophyll cleavage, PSII–LHCII supercomplex disintegration seems to be a prerequisite for the separate degradation of PSII and LHCII complexes. The course of senescence is characterized by a significant loss of thylakoid proteins of the different complexes. The proteins that have been described as being degraded from the PSII–LHCII supercomplex are PSII reaction centre D1 and D2 (Prakash *et al.*, 2001; Guiamét *et al.*, 2002; Krupinska *et al*., 2012; Nath *et al.*, 2013; Yamatani *et al.*, 2013; Li *et al.*, 2017a), chlorophyll–protein complex CP43 and CP47, cytochrome *b*_559_ apoprotein and Mn-stabilizing protein at the core complex of PSII (Guiamét *et al.*, 2002; Tang *et al.*, 2005; Krupinska *et al*., 2012; Yamatani *et al.*, 2013), CP29, CP26, and other proteins of the LHCII complex (Prakash *et al.*, 2001; Guiamét *et al.*, 2002; Tang *et al.*, 2005; Krupinska *et al*., 2012; Nath *et al.*, 2013), and PSII-associated protein PsbS (Zienkiewicz *et al.*, 2012) (Fig. 2). PetD and Rieske Fe–S protein of the cytochrome *b*_6_*f* complex also show high levels of degradation during senescence (Prakash *et al.*, 2001; Guiamét *et al.*, 2002; Krupinska *et al.*, 2012; Nath *et al.*, 2013; Li *et al.*, 2017a). In contrast, PSI activity seems to be stable until the final phases of leaf senescence, declining sharply thereafter and showing degradation of proteins of the PSI core and reaction centre and the LHCI complex (Prakash *et al.*, 2001; Guiamét *et al.*, 2002; Krupinska *et al.*, 2012; Nath *et al.*, 2013). The ATPase complex remains stable throughout senescence and shows symptoms of degradation only at the end of the process (Prakash *et al.*, 2001; Guiamét *et al.*, 2002; Nath *et al.*, 2013). Finally, the electron transport carrier plastocyanin, which acts between the cytochrome *b*_6_*f* complex and the PSI–LHCI supercomplex, appears also as a target of chloroplast degradation during senescence (Chassin *et al.*, 2002; Zienkiewicz *et al.*, 2012; Tamary *et al.*, 2019).

The degradation of stromal proteins during senescence (Fig. 2) allows the reutilization of their amino acids as a source of nitrogen in sink tissues. Rubisco, the enzyme that catalyses CO_2_ fixation and initiates carbon assimilation via the Calvin–Benson cycle, contributes up to 50% of the soluble protein and up to 30% of the total leaf nitrogen in C_3_ plants, thus being a key protein to ensure nutrient mobilization and a clear target for chloroplast degradation (Feller *et al.*, 2008). During abiotic stress-induced senescence, the cleavage of other enzymes of the Calvin–Benson cycle has also been observed, such as phosphoribulokinase, Rubisco activase, and phosphoglycolate phosphatase. In addition, the degradation of enzymes of the nitrogen assimilation pathway, such as nitrite reductase, glutamine synthetase (GS) and glutamate synthase (ferredoxin GOGAT), has also been observed (Feller *et al.*, 2008) (Fig. 2).

### Lipids

Due to their structural function as constituents of the membrane network of the chloroplast, lipids are critical for chloroplast dismantling. During natural or methyl jasmonate (MeJA)-induced senescence, 13-lipoxygenase, which is localized at the plastid envelope, catalyses the dioxygenation of unsaturated membrane fatty acids, thereby facilitating the formation of disruptions in the envelope that provoke release of stromal components (Springer *et al.*, 2016). The galactolipids MGDG and digalactosyl diacylglycerol (DGDG) are major lipid components of thylakoid membranes and play crucial roles in the structure and stability of photosynthetic complexes (Webb and Green, 1991; Hölzl and Dörmann, 2019). Thus, galactolipids also represent main targets of chloroplast degradation (Fig. 2). DGDG degradation is catalysed by alkaline α-galactosidase during leaf senescence (Lee *et al.*, 2009) and phospholipase Dδ was proposed to participate in the hydrolysis of phosphatidylcholine to phosphatidic acid (Jia *et al.*, 2013; Jia and Li, 2015). In addition, two ABA-induced plastid glicerolipid A_1_ lipases, PLIP2 and PLIP3, have been proposed to participate in MGDG and phosphatidylglycerol degradation, respectively. These lipases participate also in JA biosynthesis, and thus connect the signalling pathways of two senescence-related hormones (ABA and MeJA) (Wang *et al.*, 2018). Up to 68 putative lipases have been identified the expression of which increases during senescence (Troncoso-Ponce *et al.*, 2013), though their role in the process remains to be elucidated.

### Starch

Photosynthesis-related transitory starch is mainly degraded at night inside the chloroplast by amylases, but extraplastidic events have also been described. Large starch granules (1–2 µm length) are broken down into small granules (<1 µm length) before being delivered in selective cargo vesicles, the SSGL bodies (Wang *et al.*, 2013).

## Intra- and extraplastid events in chloroplast dismantling

Chloroplast dismantling is a complex process that requires the coordination of intra- and extraplastid events. The process of chlorophyll breakdown, which is started inside chloroplasts in senescent leaves, is completed by the release of non-fluorescent chlorophyll catabolites that are finally degraded in vacuoles (Fig. 3). The phenotypes of stay-green mutants suggest that overall chlorophyll breakdown occurs inside intact plastids; in particular, the enzymatic reactions converting the different green chlorophyll catabolites into the first colourless by-products are localized inside, whereas the metabolism of non-phototoxic colourless catabolites takes place outside the organelle (Hörtensteiner and Kräutler, 2011). Much effort have been dedicated to setting up a topographical model of the localization of the chlorophyll catabolic enzymes based on proteomics analyses and protein fusions with reporter fluorescent proteins. These studies showed the localization of NON-YELLOW COLORING1-LIKE (NOL) chlorophyll *b* reductase and pheophorbide *a* oxygenase at the inner side of the plastid envelope, pheophytinase and red chlorophyll catabolite reductase in the stroma and NON-YELLOW COLORING1 (NYC1) chlorophyll *b* reductase in thylakoid membranes (reviewed by Hörtensteiner and Kräutler, 2011). In addition, interactions between partners were identified, for example between pheophorbide *a* oxygenase and red chlorophyll catabolite reductase (Pruzinská *et al.*, 2007), NYC1 and NOL chlorophyll *b* reductase (Sato *et al.*, 2009), or even connecting all the chlorophyll catabolic enzymes to LHCII (Sakuraba *et al.*, 2012; Shimoda *et al.*, 2016).

**Fig. 4.**
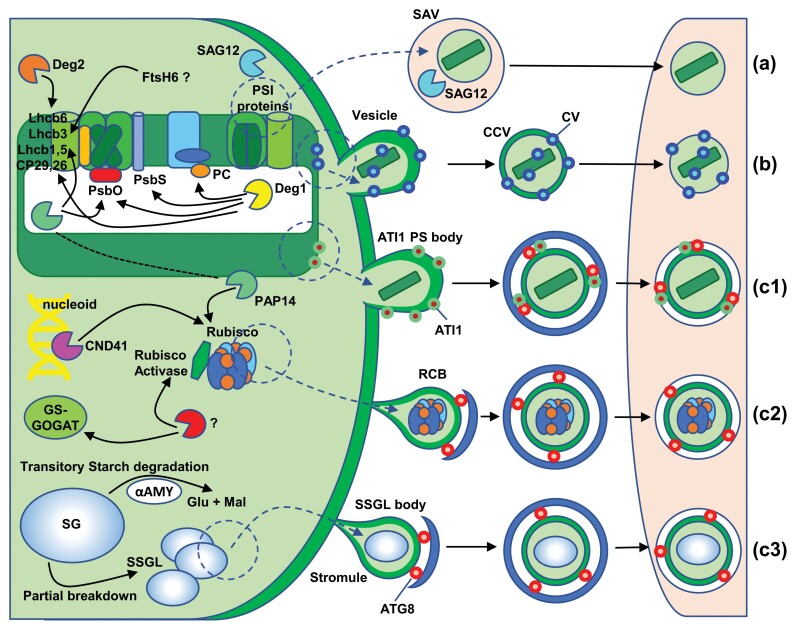
Intra- and extraplastid events of chloroplast dismantling during senescence and stress. Intraplastid events (left side) include the disassembly and cleavage of different thylakoid membrane proteins, catalysed by Deg1, Deg2, FtsH6, and HvPAP14 proteases, the proteolysis of Rubisco and other stroma proteins catalysed by CND41 and HvPAP14 proteases, and the cleavage of starch granules (SG) to small starch granules (SSG) and formation of small starch granule-like (SSGL) bodies. Extraplastid events (right side) include the different pathways for chloroplast degradation: senescence associated vacuoles (SAV) (a), chloroplast vesiculation (CV) (b), and chlorophagy (c). Three types of piecemeal chlorophagy are distinguished: ATG8-interacting protein 1-positive (ATI1 PS) body (c1), Rubisco containing bodies (RCB) (c2), and SSGL body (c3). ATI1 PS, RCB, and SSGL bodies are removed by macroautophagy, an ATG-dependent piecemeal chlorophagy through autophagosomes (blue double-membrane vesicle) marked by ATG8 (red circles). SAV and CV-containing vesicles (CCV) are degraded by microautophagy (ATG-independent pathways). αAMY, amylase; GS-GOGAT, glutamine synthetase–glutamate synthase.

Concerning intraplastid protein degradation, more than 20 chloroplast protein-degrading enzymes have been identified using a combination of biochemical, genetic, and proteomic approaches. Though it is considered that these enzymes are mainly involved in functions of housekeeping, protein quality control and maintenance of homeostasis (Nishimura *et al.*, 2017), some of them are up-regulated during leaf senescence (Roberts *et al.*, 2012), which suggests a specific role of these proteases in chloroplast dismantling. It is well known that the degradation of PSII reaction centre D1 protein after photoinhibition is mediated by the concerted action of Deg and FtsH proteases. Deg proteases are ATP-independent serine-endoproteases of which five types have been identified in chloroplasts: Deg1, Deg5, and Deg8 are localized at the luminal side of thylakoid membranes, and Deg2 and Deg7 are peripherally attached to the stromal side. Deg2 and Deg7 participate in the cleavage of photodamaged D1 protein between helices D and E (the stromal DE loop). In contrast, Deg1, Deg5, and Deg8 act on the CD loop linking helices C and D of the D1 protein at the luminal side. Fragments of D1 generated by the action of Deg proteases are then substrates for FtsH, a membrane-bound, ATP-dependent matalloprotease that performs a processive degradation (Kato *et al.*, 2012; Yoshioka-Nishimura, 2016). The process of D1 degradation and subsequent re-synthesis is part of the normal damage–repair cycle of PSII, and thus it was proposed that the net loss of PSII during senescence is caused by the accumulation of oxidative damage to a degree that exceeds the capacity of the repair system (Krieger-Liszkay *et al.*, 2019). Deg1 participates in additional degradative processes (Fig. 4), including the cleavage of LHCII antenna proteins CP29 and CP26, the PSII-associated PsbS (CP22) protein in Arabidopsis (Zienkiewicz *et al.*, 2012) under high light stress, and PSII extrinsic subunit PsbO and the soluble electron carrier plastocyanin in response to heat stress (Chassin *et al.*, 2002).

In barley senescing leaves a C1A-type cysteine protease, HvPAP14, associated to thylakoid membranes is activated by cleavage of the inhibitory pro-peptide at low pH at the luminal side of thylakoids. Once active, HvPAP14 has been shown to participate in the degradation of the oxygen-evolving complex protein PsbO and the LHCII proteins Lhcb1 and Lhcb5 (Frank *et al.*, 2019) (Fig. 4). Mature HvPAP14 might leave the thylakoid lumen and bind to the stromal side of thylakoid membranes to participate in the degradation of stromal proteins (Frank *et al.*, 2019). Deg2 protease was shown to participate in the degradation of the photosystem II light-harvesting protein Lhcb6 apoprotein in response to abiotic stresses, such as high salt, desiccation, wounding, heat, cold, and high irradiance (Luciński *et al.*, 2011). Similarly, FtsH6 was proposed to participate in the cleavage of LHCII protein Lhcb3 under high light or during senescence (Fig. 4). FtsH6 is an ATP-dependent, zinc-metalloprotease activated by the elimination of extrinsic factors yet to be identified (Zelisko *et al.*, 2005), but this result was not confirmed by *in vivo* assays, and has been questioned (Wagner *et al.*, 2011).

The degradation of stromal proteins occurs by a mixture of intra- and extraplastid events. For example, the degradation of Rubisco, Rubisco activase, and GS is initiated inside the chloroplast (Feller *et al.*, 2008; Lee *et al.*, 2013), but is completed by autophagy-related extraplastid degradation pathways (Lee *et al.*, 2013). CND41, a senescence-associated aspartic protease localized to the chloroplast nucleoid, has been shown to participate in Rubisco degradation in senescent leaves of tobacco (Kato *et al.*, 2004) (Fig. 4). As mentioned above, once fixed to the stromal side of the thylakoid membrane, mature HvPAP14 participates in the partial cleavage of the large subunit of Rubisco, provoking the accumulation of a 44-kDa cleavage product inside the chloroplast (Frank *et al.*, 2019), which is finally degraded in the vacuole (Martínez *et al.*, 2008). Finally, plastoglobules, which, as discussed above, play a relevant role in pigment and fatty acid catabolism, are degraded via intra- and extraplastid events (Fig. 3). The progressive lack of electron density shown by plastoglobules suggests that a pathway for their degradation may occur within the chloroplast (Liu, 2016), but most plastoglobules are secreted outside the organelle, either by direct exposure at the plastid surface or by protrusion of plastoglobule-containing vesicles (van Doorn and Prisa, 2014; Liu, 2016). Once in the cytosol, plastoglobules or plastoglobule-containing vesicles are engulfed by the central vacuole to undergo degradation (Liu, 2016) (Fig. 3). A similar mechanism of microlipophagy has been described for the degradation of lipid droplets during starvation-induced stress (Fan *et al.*, 2019).

Despite the intraplastid selective cleavage events, the bulk of chloroplast degradation occurs outside the organelle (Ishida *et al.*, 2014; Xie *et al.*, 2015; Otegui, 2018; Zhuang and Jiang, 2019). At least three main extraplastid pathways for chloroplast dismantling can be distinguished (Fig. 4): (i) senescence-associated vacuoles (SAVs), (ii) chloroplast vesiculation (CV) pathway, and (iii) chlorophagy, a type of autophagy in which autophagosomes selectively sequester plastid components or entire chloroplasts. SAVs are single-membrane acidic compartments of around 0.8–1 µm, defined as vacuoles due to the presence of a vacuolar H^+^ pyrophosphatase, that develop during leaf senescence (Otegui *et al.*, 2005) (Fig. 4, a). SAVs have a high content of proteolytic enzymes, though the most characteristic marker of this type of vacuole is the cysteine protease SAG12 (Otegui *et al.*, 2005; Carrión *et al.*, 2013). During leaf senescence there is a selective transfer of chloroplast components to SAVs. These include stromal proteins, such as GS II and the large subunit of Rubisco (Martínez *et al.*, 2008), and proteins from the thylakoid membranes, such as the PSI chlorophyll-binding protein PsaA and the light-harvesting complex proteins Lhca 1–4 (Gomez *et al.*, 2019). Interestingly, D1 or PSII chlorophyll-binding proteins are not degraded via SAVs, suggesting the participation of this pathway exclusively in the degradation of stroma and PSI proteins and their associated chlorophylls of the thylakoid membranes during leaf senescence, but not of PSII components (Martínez *et al.*, 2008; Gomez *et al.*, 2019). The CV pathway (Fig. 4, b) is operative in senescing leaves and in response to abiotic stress. CVs, which are induced in these conditions, are targeted to the chloroplast, and interact with proteins localized in the thylakoid, the stroma, and the envelope. After destabilizing the chloroplast membrane system, CVs promote the formation of vesicles, CV-containing vesicles, which bud from the chloroplast into the cytoplasm and are then delivered to vacuoles (Fig. 4 (b); (Wang and Blumwald, 2014).

The third pathway is chlorophagy of which two types can be distinguished: whole organelle and piecemeal chlorophagy. Whole organelle chlorophagy is observed in leaves undergoing senescence induced by dark (Wada *et al.*, 2009) or UV-B/high visible light-promoted photodamaging radiation (Izumi *et al.*, 2017). Under these conditions, large atypical autophagosomes (>1 µm length) are formed around damaged chloroplasts for selective delivery to vacuoles in wild type plants but not in autophagy deficient mutants (Wada *et al.*, 2009; Izumi *et al.*, 2017). Unlike whole organelle chlorophagy, piecemeal chlorophagy is characterized by the budding of vesicles from plastids that are then delivered to vacuoles for digestion. Selective cargo determines the three types of piecemeal chlorophagy: ATG8-INTERACTING PROTEIN 1-positive (ATI1-PS) bodies (Fig. 4, c1), RCBs (Fig. 4, c2), and SSGL bodies (Fig. 4, c3). ATI1-PS bodies (around 50–100 nm) contain chloroplast proteins originating from the stroma, the thylakoid, or the envelope but appear not to participate in Rubisco remobilization (Michaeli *et al.*, 2014). The key factor in ATI1-PS body formation is ATI1, a protein able to interact with plastid proteins and ATG8. ATI1-PS bodies bud directly from the surface of plastids into the cytoplasm in an ATG-independent manner being, however, the ATG machinery needed for the release from the cytoplasm to the vacuole. Their main function is the removal of chloroplast components under salt stress and oxidative damage conditions (Michaeli *et al.*, 2014). RCBs (Fig. 4, c2) are observed in naturally senescing leaves of wheat as double membrane vesicles containing GS and small and large subunits of Rubisco, but not thylakoid proteins (Chiba *et al.*, 2003). These bodies are formed in chloroplast projections or stromules (Spitzer *et al.*, 2015), released into the cytoplasm through an ATG-dependent autophagic process (Ishida *et al.*, 2008), and mobilized to vacuoles for degradation via the endosomal CHARGED MULTIVESICULAR BODY PROTEIN1 (Spitzer *et al.*, 2015). RCB-derived chlorophagy allows nutrient remobilization under energy-limiting conditions, such as natural senescence or dark-induced carbon starvation (Ishida *et al.*, 2014). Finally, SSGL body-derived chlorophagy (Fig. 4, c3) seems to participate in the transitory starch degradation at night by releasing neutral sugars such as glucose and maltose to support respiration and metabolism (Wang *et al.*, 2013). SSGL bodies bud off also from stromules and are released into the cytoplasm to be then delivered to vacuoles in an ATG-dependent pathway. This pathway shows similarities with the RCB pathway, but selectively carries small starch granules (Wang *et al.*, 2013).

## Conclusion

At early stages of plant development, chloroplast biogenesis is encompassed by cotyledon greening and the development of true leaves. Then, the function of chloroplasts as factories of sugars, amino acids, lipids, and other metabolic intermediates is essential to support plant growth and development, hence making possible the autotrophic lifestyle of plants. Moreover, chloroplasts have the important activity of sensing external environmental conditions that allows the harmonization of the growth of the different plant organs. Nevertheless, stressful environmental conditions may imbalance ROS production and scavenging, which generates oxidative stress and potentially may cause damage to the organelle. These conditions trigger mechanisms of chloroplast degradation, which have the function of avoiding the deleterious effects of damaged chloroplasts on cell viability. In addition, leaf senescence is a complex genetic programme that allows the remobilization and recycling of leaf components to support the growth of sink tissues, and chloroplasts constitute an important source of carbon and nitrogen in this recycling process. Therefore, chloroplast dismantling is an essential process of plant development and adaptation to stressful environmental conditions. Chloroplast dismantling is a rather complex process that involves both intra- and extraplastid events, which may be highly interconnected and tightly regulated. In this review, we have described the most significant morphological and biochemical features that characterize chloroplast dismantling in senescing leaves and in response to environmental stress such as excess light or UV/B radiation. The chloroplast-to-gerontoplast transition is characterized by morphological changes and deep structural rearrangements of the membrane network and pigment–protein supercomplexes. The sequence of events and the enzymes participating in the degradation of chloroplast components are well known and have been described in conjunction with the extraplastidial pathways operating in the process. Finally, structural analyses showing chloroplast instability in Arabidopsis mutants impaired in the redox regulatory network suggest the participation of chloroplast redox homeostasis in the degradation of the organelle. The molecular basis of redox regulation of chloroplast dismantling is still poorly known and deserves more attention in the future.
